# Supplementary material for: Extending the use of the World Health Organisations’ water sanitation and hygiene assessment tool for surveys in hospitals – from WASH-FIT to WASH-FAST
Source: PLoS One. 2019 Dec 16;14(12):e0226548. doi: 10.1371/journal.pone.0226548 (PMC6913973; doi:10.1371/journal.pone.0226548)
Supplement: S2 File — (PDF) [file pone.0226548.s002.pdf]

Semi-Structured Interview Guide for Hospital Managers and Frontline Healthcare Workers

Closed Questions

Interviewee code	
Institution	______________
Position / Role	______________
Grade (where applicable)	______________
Age	______________
Gender	______________
Highest education / training	______________
Years of experience in Infection Prevention and Control ¥	______________
Membership to relevant organisations	______________

Core competencies in infection prevention and control ¥

(Tick the option that best defines the interviewee's role)

Management / Supervision ☐ Frontline contact, service or care ☐

Comments:

1. What IPC guidelines are available in this hospital? In your view, what guidelines/documents do you feel have affected the way you practice in this hospital?
   Step through all general and domain-specific policy documentation and enquire about potential additional sources of information. >>

2. Do you have an IPC team? When did they last meet?
   Ask who are members, when it meets, what roles and responsibilities are? >>

3. How are doctors, nurses and other healthcare workers trained for IPC at this hospital? How are they trained on the new guidelines? As a student?
   Ask what percentage of staff have received; if renewed; who gave the training.>>

4. Is there a specific IPC budget? How much is it annually?
   Investigate annual budget setting, how funds are allocated in general. Is IPC only funded as a management area by external sources? >>

5. What are the main IPC problems you experience at the hospital level?
   <<Step into each one. Ask for examples, details on what the problems are and how they're dealt with>>
   Probes
   Following the available guidelines by the clinicians?
   Funding
   Lack of supply or poor quality Equipment for IPC activities e.g. gloves, masks

6. What do you think patients know about IPC?
   <<Exploratory – looking for background. If they use phrases like 'ignorant', ask what they mean by this. If they differentiate between themselves and the patients, ask why nurses/doctors are different.>>

   <<Explain AMR – infections becoming resistant to drugs – you know about Multi Drug Resistant TB, but happening to a lot of infections. Very difficult to treat – running out of antibiotics. IPC is one way of dealing with AMR, but we're also interested in how you use antibiotics…

7. Have you heard of antibiotic stewardship? (yes – move to 8; no – explain ABS is where we're careful we don't use antibiotics for infections that don't need them, or use the wrong antibiotics, or don't make sure patients finish their course).

8. Are there any guidelines or training for ABS (***correct and rational use of antibiotics***)? Is this dealt with as part of IPC?
   By training this may include CME, seminars and external training
   <<Explore any differences. Be prepared to explain what ABS is. >>

9. How are doctors, nurses and other healthcare workers trained for ABS at this hospital? How are they trained on the new guidelines? As a student?
   Ask what percentage of staff have received; if renewed; who gave the training.>>

10. Who sets policy for ABS in the hospital? What guidelines, if any are they currently using for prescription of the antibiotics? How are you able to identify if

antibiotics are not working? How is antibiotic resistance dealt with in this hospital?
Identify documents and guidelines in actual use first. Then ask if they are aware of national guidelines etc.

11. What do you think patients should know about antibiotics? Do you have patients that have preferred antibiotics? Do they know it's important they should finish the course of medication? Do they sometimes stop and start? How do you get them to finish the course of medication?

<<*As above, focus on any accusations of 'ignorance' or 'lack of education'. Ask what this means, and how doctors/nurses deal with this.* >>

12. I can imagine the workers at this hospital are very busy and have lots on their minds. How much do you think that IPC and ABS are prioritised at this hospital? Do medical staff see IPC as something important? how? why?
<<Do medical staff take responsibility for IPC? >>

(Thank the interviewee for their time, explain the project again, and inform them that they will be made aware of findings.)
