# Supplementary material for: Extending the use of the World Health Organisations’ water sanitation and hygiene assessment tool for surveys in hospitals – from WASH-FIT to WASH-FAST
Source: PLoS One. 2019 Dec 16;14(12):e0226548. doi: 10.1371/journal.pone.0226548 (PMC6913973; doi:10.1371/journal.pone.0226548)
Supplement: Attachment — Submitted filename: Response to Editors Plos.docx [file pone.0226548.s004.docx]

# KEMRI/ WELLCOME TRUST RESEARCH PROGRAMME

(Centre for Geographic Medicine Research – Coast)

**P.O.Box 230, 80108, Kilifi, Kenya	P.O.Box 43640, 00100, Nairobi, Kenya**

**Tel: (+254) 417 522535/522063/525044/525453	Tel: (+254) 20 2710672/2715160**

**Fax: (+254) 417 522390	Fax: (+254) 20 2711673**

**Email:** mmaina@kemri-wellcome.org

8^th^ July 2019

To:	The Editor

Plos One, Antimicrobial Resistance for papers

Response to the Editor: PONE-D-19-11136

We are delighted to submit this revised manuscript under the AMR call for papers. We wish to indicate that we had submitted this manuscript earlier to Plos One (PONE-D-19-11136)) but not to the AMR call for papers.

**The Editors comments** :

*The manuscript titled "Purposeful design of a survey tool to evaluate the adequacy of hospitals' water and sanitation and hygiene and allocate responsibility for action - From WASH FIT to WASH FAST" attempts to address important limitations of WASH FIT, i.e. regional and national situational analysis, monitoring and tracking etc. This is very important as it seeks to address an important gap and help in improving WASH programmes.
Unfortunately, there are serious methodological flaws. Based on the processes described in the methods section, the validity of the WASH FAST is questionable. The authors failed to demonstrate that it measures accurately what it intends to measure. The qualitative methods described under the methods sections cannot adequately validate the WASHFAST to address the weaknesses identified in WASHFIT*

**Response:**

We appreciate the feedback and views from the editors; we wish to state that our main aim was not to validate the WASH-FIT or WASH FAST in a statistical sense. The indicators that comprise the WASH FIT tool and that are used in the WASH-FAST adaptation had already undergone an extensive development process overseen by WHO and this sense the indicators have been validated by the global WASH and infection prevention and control community as now briefly described in our manuscript, Page 4.

In collaboration with WHO developers of WASH FIT, our manuscript proposes an extension of the second step of WASH FIT (i.e. the facility assessment step) into WASH Facility Survey Tool (WASH-FAST), to meet broader application needs and to facilitate comprehensive assessment of WASH in larger – more complex – secondary and tertiary healthcare facilities encompassing both outpatient and inpatient care.

Our aim was indeed to extend the use of the existing indicators to include assessment at the individual ward level and to develop an intuitive semi-quantitative scoring approach that could be employed to highlight where WASH challenges are greatest, to demonstrate variations across place that might be useful when reviewing the results of large scale monitoring exercises and potentially provide a simple means of tracking progress in addressing challenges over time. In linked work, we also aimed to demonstrate how these existing indicators may be used to assign or highlight at which level of the health systems for addressing challenges may lie.

Of note, WASH FAST builds on the same previously validated indicators and scoring system of WASH FIT but applies subsets of indicators to assess the performance of individual wards.

The aim of the qualitative interviews and the stakeholder workshops that were conducted were to ensure the views of multiple stakeholders were considered in these efforts to identify those responsible for action to improve WASH at different levels of the health system. These stakeholder workshops and interviews were also used to establish the feasibility and potential value of the WASH Facility Survey Tool (WASH-FAST) and to check that the data aggregation and visualisation methods used to demonstrate findings and their variability were appropriate and useful. We have therefore rewritten this manuscript to highlight these issues and also made the title clearer. We hope these changes will be sufficient for consideration of this manuscript.

We argue that our manuscript should be published for local, regional and national government actors to consider the extended applications that can be met around situation analysis, monitoring of WASH and intervention impact measurement if the formative elements of WASH FIT were implemented following the extended WASH FAST format. We believe the manuscript will promote increased attention to core health systems issues that influence the emergence and transmission of AMR and will resonate with many of your readers in LMICs. For this reason, we are hopeful that you will consider this revised manuscript for publication.

Thank You

Dr Michuki Maina

On behalf of the Authors
