# Supplementary material for: Extending the use of the World Health Organisations’ water sanitation and hygiene assessment tool for surveys in hospitals – from WASH-FIT to WASH-FAST
Source: PLoS One. 2019 Dec 16;14(12):e0226548. doi: 10.1371/journal.pone.0226548 (PMC6913973; doi:10.1371/journal.pone.0226548)
Supplement: Attachment — Submitted filename: Response to Reviewers_sept.docx [file pone.0226548.s005.docx]

# KEMRI/ WELLCOME TRUST RESEARCH PROGRAMME

(Centre for Geographic Medicine Research – Coast)

**P.O.Box 230, 80108, Kilifi, Kenya	P.O.Box 43640, 00100, Nairobi, Kenya**

**Tel: (+254) 417 522535/522063/525044/525453	Tel: (+254) 20 2710672/2715160**

**Fax: (+254) 417 522390	Fax: (+254) 20 2711673**

**Email:** mmaina@kemri-wellcome.org

1^st^ October 2019

To:	The Editor

Plos One, Antimicrobial Resistance for papers

**Re: PONE-D-19-19422 Extending the use of the World Health Organisations’ water sanitation and hygiene assessment tool for surveys in hospitals – from WASH-FIT to WASH FAST**

Thank you for the review and comments provided for this manuscript. We have addressed the one query raised as shown below.

**Reviewers Comments**

Reviewer #1: Dear authors,

Thank you for the opportunity to review the manuscript, “Extending the use of the World Health Organisations’ water sanitation and hygiene assessment tool for surveys in hospitals– from WASH-FIT to WASH FAST.”

For the limited national context, possibly an interesting study that possibly is suitable for the national Health Department. Lacking scientific underpinning and low applicability for an international context. No considerable new insights.

**Response**

We thank the reviewers for taking the time to review this manuscript. In response to the above comment.

Water Sanitation and Hygiene (WASH) has been highlighted as a crucial but under-resourced aspect of the fight against hospital-acquired infections and antimicrobial resistance (AMR) and policymakers’ efforts to intervene in these areas are fundamentally limited. This has been in part due to the lack of understanding of the status of the hospitals under their charge. The WASH-FIT tool is in use around the world and the World Health Organization (WHO) has promoted its use as a tool in improving water and sanitation and thus reducing infection and opportunities for AMR. As such, our extension of this important tool is highly relevant to Low- and middle-income countries considering interventions in this important area and could increase the impact of the tool wherever it is used.  This work is relevant to teams conducting research or developing interventions to improve WASH as it forms a good base to measure performance on WASH. For governments, international organizations and funders who are interested in investing in WASH activities, our work also provides a possible way to measure and monitor WASH performance.

The original WASH FIT from which we propose the extension underwent a rigorous validation process and was piloted in several countries before its eventual rollout ( Page 4/5). The 65 WASH indicators used for assessment in the tool are based on existing scientific evidence and are derived from global environmental and infection prevention and control standards (Page 5 Line 76-80). We are therefore of the view that this work indeed has major scientific underpinning. We have also highlighted this in the discussion section of the manuscript. For the reviewer’s reference, we are actively working with the WHO to ensure our findings *do* have a global impact.

**Reviewers Comments**

**Reviewer #2: Dear authors,**
Thank you for the opportunity to review your manuscript "Purposeful design of a survey tool to evaluate the adequacy of hospitals' water and sanitation and hygiene and allocate responsibility for action - From WASH FIT to WASH FAST", in which you present important limitations of WASH FIT and Adaption do WASH FAST. Firstly, I would like to congratulate you for having drafted that very relevant and interesting research. However, prior to a possible publication, I would like to share my thoughts on the manuscript:

1.) Within the Abstract the limitations of WASH-FIT should be highlighted shortly, to comprehend the need for an adaption.

2.) Please try and design the background part in a more legible way. There are too many abbreviations in quick succession which makes it hard to understand.
**Response**

Thank you for your review and feedback.

1. We have put in the abstract a sentence on the main limitations of the WASH FIT. Which are that it was mainly designed for smaller hospitals with mainly outpatient and limited inpatient care. It was also not designed as a tool to monitor or compare performance across hospitals (Page 2 Line 25-26). We have also highlighted these are other limitations more clearly in the introduction section of the manuscript. (Page 5 Line 80-96)
2. Our apologies for the several abbreviations, we have attempted to put most of them in the manuscript as full text instead. The introduction section of the manuscript has been modified to be clearer and more legible.

The first section of the introduction highlights what Water Sanitation and Hygiene(WASH) is and its importance in health (Page 4 Line 51-59).

In the second section, we describe what is known on the subject and the existing gaps (Page 4 Line 61-68).

We then proceed to highlight the introduction of the tool developed by the World Health Organization to monitor and improve WASH (Page 4 Line 69-80).

The next paragraph goes on to highlight what are the main shortcomings of the WASH FIT tool as it stands (Page 5 Line 82-98).

Using Kenya as an example we present the context in which this tool is meant to work highlighting that the health systems are more complex than envisaged in the WASH tool. (Page 6 Line 99-107).

The final paragraph highlights what our work is about by introducing a modification of the tool to meet some of the shortcomings described earlier (Page 6 Line 108-114).

Thank You

Dr Michuki Maina

On behalf of the Authors
