# Supplementary material for: Extending the use of the World Health Organisations’ water sanitation and hygiene assessment tool for surveys in hospitals – from WASH-FIT to WASH-FAST
Source: PLoS One. 2019 Dec 16;14(12):e0226548. doi: 10.1371/journal.pone.0226548 (PMC6913973; doi:10.1371/journal.pone.0226548)
Supplement: Attachment — Submitted filename: Response to ReviewersMainaetalNov.docx [file pone.0226548.s006.docx]

# KEMRI/ WELLCOME TRUST RESEARCH PROGRAMME

(Centre for Geographic Medicine Research – Coast)

**P.O.Box 230, 80108, Kilifi, Kenya	P.O.Box 43640, 00100, Nairobi, Kenya**

**Tel: (+254) 417 522535/522063/525044/525453	Tel: (+254) 20 2710672/2715160**

**Fax: (+254) 417 522390	Fax: (+254) 20 2711673**

**Email:** mmaina@kemri-wellcome.org

13^th^ November 2019

To:	The Editor

Plos One, Antimicrobial Resistance for papers

**Re: PONE-D-19-19422 Extending the use of the World Health Organisations’ water sanitation and hygiene assessment tool for surveys in hospitals – from WASH-FIT to WASH FAST**

Thank you for the review and comments provided for this manuscript. We have made the changes as suggested.

**The Reviewers comments** :

1. Dear authors thank you for the opportunity to review the manuscript „Extending the use of the World Health Organisations’ water sanitation and hygiene assessment tool for surveys in hospitals – from WASH-FIT to WASH-FAST”.

The need to comply with elementary hygiene rules is evident and requires no scientific investigation. The potential application of the "tool" wash-fast seems to be an inappropriate way to counteract non-compliance with elementary hygiene rules.

**Response:**

Thank you taking time to review this manuscript and for the comment. We agree with the reviewer that it requires no scientific investigation to assert whether proper WASH is crucial– but what we are trying to do is to provide a structured tool that allows hospitals, Ministry of Health and others health agencies to evaluate compliance with hygiene rules and to summarise findings on compliance. The need for such monitoring tools is recognised by WHO and the global community who developed WASH-FIT – and the findings of their global survey of smaller facilities has been important in identifying the gap between self-evident WASH rules and what is actually in place. This evidence is being used to advocate for more support to WASH implementation.

Monitoring and reporting is an essential step to addressing and improving compliance, and an essential step for implementation of any sound public health intervention. Mechanisms for standardized monitoring and accountability of WASH in Low and Midddle income countries are lacking and we therefore understand this is an important gap that needs addressing. What we have done in our work done is develop this monitoring / compliance assessment further so it is better suited for hospitals with multiple inpatient units but in addition provide a form of accountability which has been lacking in the efforts to improve WASH.

2. **Reviewers Comment**

Thank you for your thorough revision. However, I noticed some more aspects that could again improve the manuscript: Your conclusion section does not provide enough sufficient information. In my opinion, its length is way too short and you should claim, why WASH-FAST has an international context more detailed. Further, the main differences and improvements between WASH-FIT and WASH-FAST should be highlighted in one sentence.

**Response**

Thank you for the comments. We have modified the conclusion to highlight the main differences between the WASH-FIT and WASH-FAST. In addition, we have summarized why this tool is relevant to hospitals, governments and international agencies. Page 20 Line 441-450.

Thank You

Dr Michuki Maina

On behalf of the Authors
